# High cell surface expression and peptide binding affinity of HLA-DQA1*05:03, a susceptible allele of neuromyelitis optica spectrum disorders (NMOSD)

**DOI:** 10.1038/s41598-021-04074-1

**Published:** 2022-01-07

**Authors:** Shohei Beppu, Makoto Kinoshita, Jan Wilamowski, Tadahiro Suenaga, Yoshiaki Yasumizu, Kotaro Ogawa, Teruyuki Ishikura, Satoru Tada, Toru Koda, Hisashi Murata, Naoyuki Shiraishi, Yasuko Sugiyama, Keigo Kihara, Tomoyuki Sugimoto, Hisashi Arase, Daron M. Standley, Tatsusada Okuno, Hideki Mochizuki

**Affiliations:** 1 Department of Neurology, Graduate School of Medicine, Osaka University, 2-2 Yamadaoka, Suita, Osaka 565-0871 Japan; 2 Department of Genome Informatics, Genome Information Research Center, Research Institute for Microbial Diseases, Osaka University, 3-1 Yamadaoka, Suita, Osaka 565-0871 Japan; 3 Department of Microbiology, Fukushima Medical University, 1 Hikariga-oka, Fukushima, Fukushima 960-1295 Japan; 4 Department of Experimental Immunology, WPI Immunology Frontier Research Center, Osaka University, 3-1 Yamadaoka, Suita, Osaka 565-0871 Japan; 5 Integrated Frontier Research for Medical Science Division, Institute for Open and Transdisciplinary Research Initiatives (OTRI), Osaka University, 2-2 Yamadaoka, Suita, Osaka 565-0871 Japan; 6 Graduate School of Data Science, Shiga University, 1-1-1 Banba, Hikone, Shiga 522-8522 Japan; 7 Department of Immunochemistry, Research Institute for Microbial Diseases, Osaka University, 3-1 Yamadaoka, Suita, Osaka 565-0871 Japan; 8 Laboratory of Immunochemistry, WPI Immunology Frontier Research Center, Osaka University, 3-1 Yamadaoka, Suita, Osaka 565-0871 Japan; 9 Systems Immunology Laboratory, WPI Immunology Frontier Research Center, Osaka University, 3-1 Yamadaoka, Suita, Osaka 565-0871 Japan

**Keywords:** Autoimmunity, Neuroimmunology

## Abstract

Neuromyelitis optica spectrum disorder (NMOSD) is a relapsing autoimmune disease characterized by the presence of pathogenic autoantibodies, anti-aquaporin 4 (AQP4) antibodies. Recently, HLA-DQA1*05:03 was shown to be significantly associated with NMOSD in a Japanese patient cohort. However, the specific mechanism by which HLA-DQA1*05:03 is associated with the development of NMOSD has yet to be elucidated. In the current study, we revealed that HLA-DQA1*05:03 exhibited significantly higher cell surface expression levels compared to other various *DQA1* alleles, and that its expression strongly depended on the amino acid sequence of the α1 domain, with a preference for leucine at position 75. Moreover, in silico analysis indicated that the HLA-DQ encoded by HLA-DQA1*05:03 preferentially presents immunodominant AQP4 peptides, and that the peptide major histocompatibility complexes (pMHCs) are more energetically stable in the presence of HLA-DQA1*05:03 than other *HLA-DQA1* alleles. In silico 3D structural models were also applied to investigate the validity of the energetic stability of pMHCs. Taken together, our findings indicate that HLA-DQA1*05:03 possesses a distinct property to play a pathogenic role in the development of NMOSD.

## Introduction

Neuromyelitis optica spectrum disorder (NMOSD) is a relapsing autoimmune disease that causes inflammation in the brain, spinal cord, and optic nerve, and it mainly affects middle-aged women^1^. In 2004, Lennon et al. discovered a specific autoantibody, NMO-IgG, in patient serum^2^, and it has subsequently been learned that the target antigen of NMO-IgG is aquaporin 4 (AQP4), the main water channel protein of the central nervous system that is densely expressed in astrocyte endfeet. Taken together, the main pathology of NMOSD is now recognized to be astrocytopathy caused by anti-NMO-IgG, currently referred to as anti-AQP4 antibodies.

Despite these important breakthroughs, little has been known about the genetic factors of NMOSD. In 2018, a whole-genome sequence study conducted on 215 NMOSD cases and 1244 controls of European ancestry identified two independent signals associated with NMO-IgG positivity in the major histocompatibility complex (MHC) regions, one of which is located in the *HLA-DQA1* gene^3^. Subsequently, in 2019, Ogawa et al*.* performed HLA genotyping using next-generation sequencing on 31 Japanese NMOSD patients and 429 healthy controls, and found that HLA-DQA1*05:03 had a significant association with NMOSD^4^.

The human leukocyte antigen (HLA) that are encoded by the MHC gene complex in humans can be classified into class I and class II proteins. Class II molecules, to which HLA-DQ belongs, is a transmembrane protein composed of an α chain consisting of α1 and α2 domains, and a β chain consisting of β1 and β2 domains, and is generally expressed on antigen-presenting cells (APCs). MHC class II binds to a peptide taken up by endocytosis via the peptide binding groove and translocates to the cell surface, thereby transmitting signals to naive CD4+ T cells to promote their differentiation into effector helper T cells.

Although the main pathology of NMOSD is astrocytopathy caused by anti-AQP4 antibodies, it has also been reported that T cells play a pivotal role in NMOSD pathogenesis. Varrin-Doyer et al*.* reported that NMOSD patients had a higher frequency of AQP4-reactive T cells than healthy controls, and that these cells exhibited Th17 polarization^5^. In addition, it has been reported that anti-AQP4 antibodies belong to the IgG1 subtype, a T cell-dependent Ig subclass^6,7^, and that their production requires AQP4 reactive CD4+ helper T cells.

From these observations, it is reasonable to theorize that the presentation of AQP4 peptide to CD4+ T cells by MHC class II promotes their differentiation from naive T cells to effector T cells, thus subsequently promoting anti-AQP4 antibody production. It can further be hypothesized that NMOSD patients with HLA-DQA1*05:03 will undergo this process more efficiently.

In this current study, we evaluated the cell surface expression of HLA-DQA1*05:03, as well as the binding of *HLA-DQA1* alleles to AQP4 peptides and the energetic stability of the resulting complexes, in order to test our above-mentioned hypotheses. Our findings indicate the possibility that HLA-DQA1*05:03 contributes to the pathogenesis of NMOSD through preferentially presenting AQP4 peptides by several distinct mechanisms.

## Results

### HLA-DQA1*05:03 is highly expressed on the cell surface

**Figure 1:**
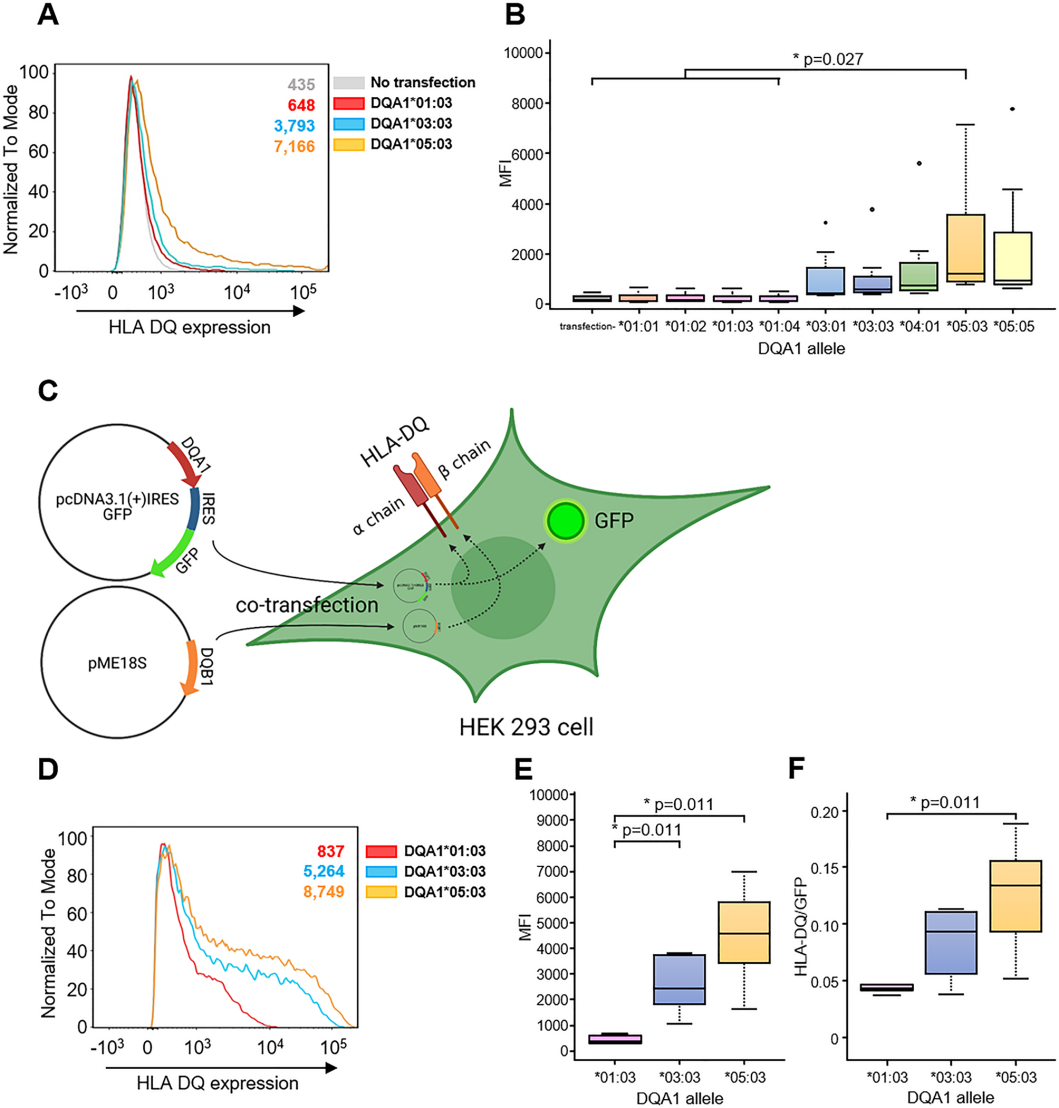
Expression of HLA-DQ molecules in HEK293 cells. (**A**) Representative cell-surface expression of HLA-DQ molecules in HEK 293 cells which were transiently co-transfected with *HLA-DQA1* and *HLA-DQB1* expression vector. HLA-DQA1*01:03 (red), DQA1*03:03 (blue) and DQA1*05:03 (yellow) were selected as the *HLA-DQA1* expression vectors and the paired *HLA-DQB1* expression vector were all HLA-DQB1*03:01. HEK293 cells that were not transfected with any *HLA* expression vector are shown as a negative control (gray). The numbers on the upper right of the graph denote mean fluorescence intensity (MFI) of HLA-DQ of the HEK293 cells. (**B**) Summary of HLA-DQ expression with nine *DQA1* expression vectors. HEK293 cells which were not transfected with any *HLA* expression vector are shown as “transfection-”. (**C**) Schema of co-transfection of pcDNA3.1 (+) IRES GFP plasmid vector that co-expresses GFP and *DQA1* allele, and pME18S plasmid vector that expresses *DQB1* allele into HEK293 cells. At 48-eight hours after transfection, the cell surface HLA-DQ and cellular GFP level were analyzed by flow cytometry. This schema was created with BioRender.com. (**D**) Representative cell-surface expression of HLA-DQ molecules in GFP-positive HEK 293 cells. GFP expression vector was constructed for HLA-DQA1*01:03 (red), DQA1*03:03 (blue) and DQA1*05:03 (yellow). All *HLA-DQB1* expression vector was DQB1*03:01. The numbers on the upper right of the graph denote the MFI of HLA-DQ of the GFP + HEK293 cells transfected with each *DQA1* allele. (**E**) HLA-DQ expression with three *DQA1* expression vectors in GFP positive HEK293 cells. (**F**) The MFI (HLA-DQ)/MFI (GFP) ratio of GFP positive HEK293 cells for each *HLA-DQ* allelic pair.

Since the relationship between HLA cell surface expression level and disease susceptibility has been clarified in several recent reports, we started by revealing the characteristics of cell surface expression levels of the HLA-DQ molecule encoded by HLA-DQA1*05:03. To investigate whether the cell surface expression levels of HLA-DQ molecules depend on haplotypes, we transiently transfected various *HLA-DQ* alleles into HEK293 cells, which lack endogenous HLA class II expression, and measured the cell surface expression level of the HLA-DQ molecule by flow cytometry. As for the *HLA-DQ* expression vectors, we selected nine *HLA-DQA1* alleles (DQA1*01:01, DQA1*01:02, DQA1*01:03, DQA1*01:04, DQA1*03:01, DQA1*03:03, DQA1*04:01, DQA1*05:03 and DQA1*05:05), which encompass most *HLA-DQA1* alleles in the Japanese population, together with HLA-DQB1*03:01. HEK293 cells were transiently co-transfected with one of the *HLA-DQA1* expression vectors and the HLA-DQB1*03:01 expression vector, which is known to be able to pair with most types of *DQA1* alleles in the Japanese population. It was observed that HLA-DQ molecules encoded by HLA-DQA1*05, especially HLA-DQA1*05:03, had the highest cell surface expression level (Fig. 1A, B).

To further confirm that HLA-DQ molecules encoded by HLA-DQA1*05:03 show high cell surface expression, we used a pcDNA3.1(+) IRES GFP plasmid vector in order to express GFP as an internal control (Fig. 1C). Co-expression vectors of GFP and *HLA-DQA1* were constructed for HLA-DQA1*01:03, DQA1*03:03, and DQA1*05:03, and the HLA-DQB1*03:01 expression vector was transiently transfected in combination with these vectors. The cell surface expression level of HLA-DQ and the expression of cytosolic GFP were determined by flow cytometry. In the GFP-positive cells, the highest expression of HLA-DQ was observed in the HLA-DQA1*05:03 group among all the *HLA-DQA1* alleles examined, consistent with the previous results (Fig. 1D, E). For each *HLA-DQ* allelic pair, we also calculated the mean fluorescence intensity (MFI) of HLA-DQ relative to the MFI of GFP, which indicates the amount of cell-surface HLA normalized to GFP, and observed that HLA-DQ encoded by HLA-DQA1*05:03 had the highest value (Fig. 1F).

Since it was unclear to what extent the peptides bound to these HLA-DQ molecules affected their cell surface expression levels, we evaluated whether the cell surface expression level of HLA-DQ could be altered by binding to AQP4 peptides. We selected five immunodominant AQP4 peptides (p61–80, p91–110, p131–150, p201–220 and p211–230) that reportedly promote the activation and proliferation of T cells with APCs^5,8–11^ (Table 1). A previous study demonstrated that ectopically expressed HLA in HEK293 cells could load exogenously added epitope peptides^12^. Therefore, the transfected HEK293 cells were incubated with each of the five AQP4 peptides for 8 h^12,13^. The hierarchy of cell surface expression levels among the *HLA-DQA* alleles observed in Fig. 1 was maintained even when co-cultured with four out of the five immunodominant AQP4 peptides (Supplemental Fig. S1). These findings demonstrated that the HLA-DQ molecule encoded by the HLA-DQA1*05:03 allele had the highest cell surface expression level irrespective of the presence of immunodominant AQP4 peptides.

**Table 1:** AQP4 peptide list. Peptides with a length of 20 amino acids constituting AQP4 molecule (323 amino acids in length) was listed as overlapping by 10 amino acids.

Peptides	Amino acid sequences	Immunodominance	References
p1–20	MSDRPTARRWGKCGPLCTRE	–	
**p11**–**30**	**GKCGPLCTRENIMVAFKGVW**	**+**	^1,3,5^
p21–40	NIMVAFKGVWTQAFWKAVTA	–	
p31–50	TQAFWKAVTAEFLAMLIFVL	–	
p41–60	EFLAMLIFVLLSLGSTINWG	–	
p51–70	LSLGSTINWGGTEKPLPVDM	–	
**p61**–**80**	**GTEKPLPVDMVLISLCFGLS**	**+**	^1,3,5^
p71–90	VLISLCFGLSIATMVQCFGH	–	
p81–100	IATMVQCFGHISGGHINPAV	–	
**p91**–**110**	**ISGGHINPAVTVAMVCTRKI**	**+**	^3,5^
p101–120	TVAMVCTRKISIAKSVFYIA	–	
p111–130	SIAKSVFYIAAQCLGAIIGA	–	
p121–140	AQCLGAIIGAGILYLVTPPS	–	
**p131**–**150**	**GILYLVTPPSVVGGLGVTMV**	**+**	^1,2,4^
p141–160	VVGGLGVTMVHGNLTAGHGL	–	
p151–170	HGNLTAGHGLLVELIITFQL	–	
p161–180	LVELIITFQLVFTIFASCDS	–	
p171–190	VFTIFASCDSKRTDVTGSIA	–	
p181–200	KRTDVTGSIALAIGFSVAIG	–	
p191–210	LAIGFSVAIGHLFAINYTGA	–	
**p201**–**220**	**HLFAINYTGASMNPARSFGP**	**+**	^2,3,4^
**p211**–**230**	**SMNPARSFGPAVIMGNWENH**	**+**	^1,3,4,5^
p221–240	AVIMGNWENHWIYWVGPIIG	–	
p231–250	WIYWVGPIIGAVLAGGLYEY	–	
p241–260	AVLAGGLYEYVFCPDVEFKR	–	
p251–270	VFCPDVEFKRRFKEAFSKAA	–	
**p261**–**280**	**RFKEAFSKAAQQTKGSYMEV**	**+**	^1,4^
p271–290	QQTKGSYMEVEDNRSQVETD	–	
p281–300	EDNRSQVETDDLILKPGVVH	–	
p291–310	DLILKPGVVHVIDVDRGEEK	–	
p301–320	VIDVDRGEEKKGKDQSGEVL	–	

AQP4 amino acid sequence of the molecules refers to the UniProt database (AQP4_HUMAN P55087-1). The immunodominant AQP4 peptides are shown in bold type.

### Amino acid sequence of the α1 domain determines the expression level of HLA-DQA1*05:03 on the cell surface

**Figure 2:**
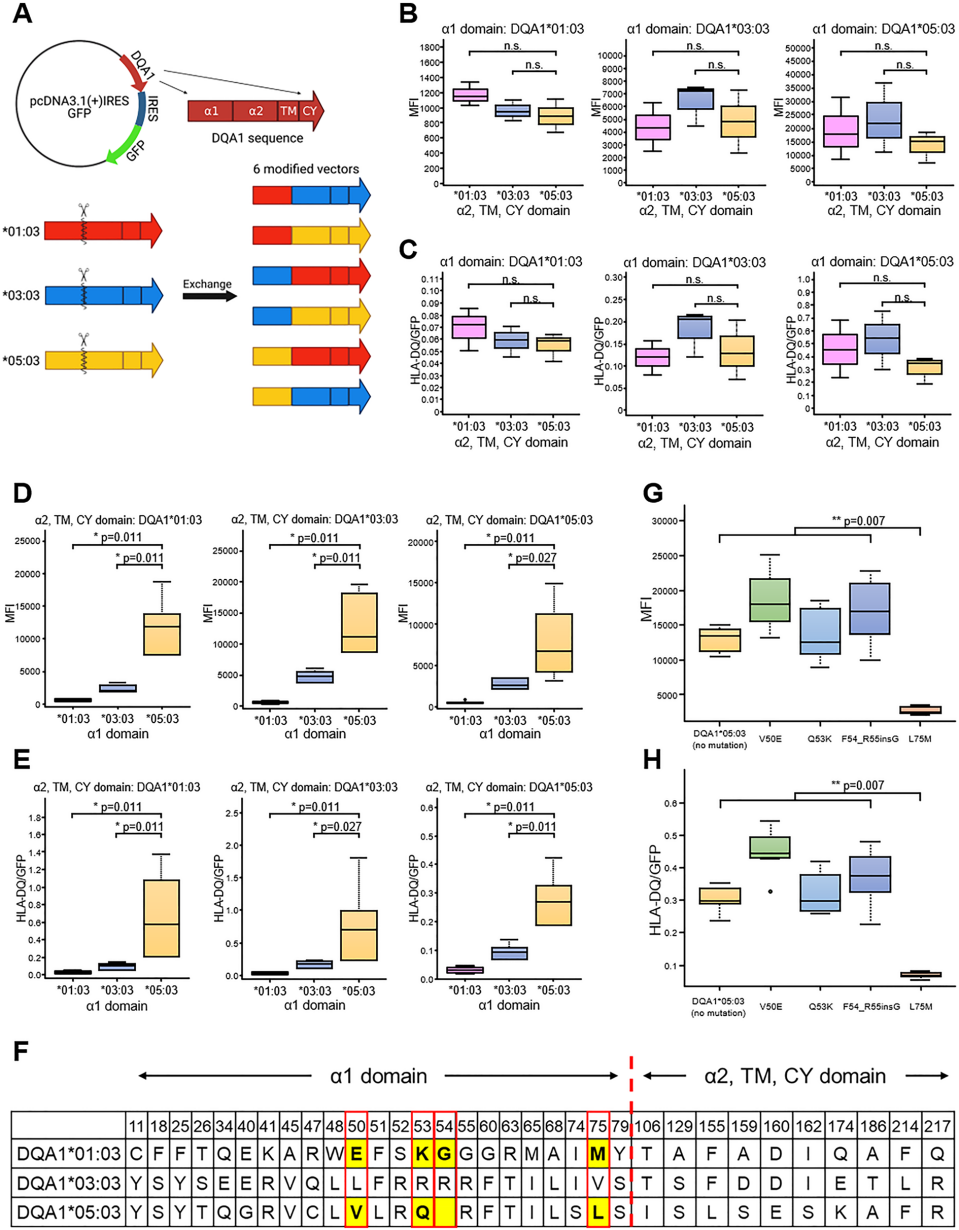
Pivotal domains that determine cell surface expression levels of HLA-DQ molecules. (**A**) Schematic representation of DQA1*01:03 (red), DQA1*03:03 (blue) and DQA1*05:03 (yellow) expression vectors and six pattern constructs with α1 domain and α2, TM and CY domain exchanged among them. This schema was created with BioRender.com. (**B**, **C**) Effect of α2 domain on HLA expression level when the α1 domain is the same. The expression level was evaluated by MFI of HLA-DQ (**B**) and MFI (HLA-DQ)/MFI (GFP) ratio (**C**) of GFP positive HEK293 cells. (**D**, **E**) Effect of α1 domain on HLA expression level when the α2 domain is the same. The expression level was evaluated by MFI of HLA-DQ (**D**) and MFI (HLA-DQ)/MFI (GFP) ratio (**E**) of GFP positive HEK293 cells. (**F**) Amino acid sequence variation between DQA1*01:03, DQA1*03:03 and DQA1*05:03. Four types of modified *HLA-DQA1* expression vectors were generated, each with a one amino acid substitution in the α1 domain sequence from HLA-DQA1*05:03 to HLA-DQA1*01:03; V50E, Q53K, F54_R55insG and L75M. (**G**, **H**) Expression of HLA-DQ molecules in HEK293 cells transfected with four types of modified vector that encodes *HLA-DQA1* sequence which has a one amino acid substitution in the α1 domain sequence from HLA-DQA1*05:03 to HLA-DQA1*01:03; V50E, Q53K, F54_R55insG and L75M. The expression level was evaluated by MFI of HLA-DQ (**G**) and MFI (HLA-DQ)/MFI (GFP) ratio (**H**) of GFP positive HEK293 cells.

Furthermore, to investigate the possibility that the hierarchy is caused by a variation in the amino acid sequence that constitutes the HLA-DQ molecule, we generated combinatorically modified *HLA-DQA1* expression vectors in which the first half of the sequences encoding α1 domains and the latter half encoding α2 domains, transmembrane regions (TM) and cytoplasmic regions (CY), were individually exchanged among HLA-DQA1*01:03, DQA1*03:03, and DQA1*05:03 alleles (Fig. 2A).

There was no significant difference in cell surface expression of HLA-DQ molecules among the modified expression vectors with the same sequence in the first half (Fig. 2B, C). On the other hand, when comparing vectors with the same sequence in the latter half, a large difference in cell surface expression was observed; when α1 domain was comprised by HLA-DQA1*05:03, the cell surface expression was the highest (Fig. 2D, E). Taken together, these data demonstrated that the amino acid sequence of α1 domain controls the difference in the cell surface expression levels of HLA-DQ molecules.

Furthermore, to determine which position in the amino acid sequence of the α1 domain was particularly important for the cell surface expression of the HLA-DQ molecule, we generated four types of modified vectors, each with one amino acid substituted in the α1 domain from those originally found in HLA-DQA1*05:03 to that in HLA-DQA1*01:03; specifically, V50E, Q53K, F54_R55insG and L75M (Fig. 2F). These four positions, all of which are located in the peptide binding cleft of HLA-DQ, were selected because various polymorphisms were observed in these positions among *HLA-DQA1* alleles. Alteration of V50E, Q53K and F54_R55insG did not result in any significant changes in the cell surface expression levels of HLA-DQ, but the mutation L75M showed a significant decrease in the cell surface expression compared to wild-type HLA-DQA1*05:03 (Fig. 2G, H).

### Immunodominant AQP4 peptides have high binding affinity with HLA-DQ molecules encoded by the HLA-DQA1*05 allele

To clarify whether HLA-DQA1*05:03, the highest risk allele of NMOSD, efficiently presents immunodominant AQP4 peptides, we compared the binding affinity of various *HLA-DQA1* alleles with both immunodominant and non-immunodominant AQP4 peptides using NetMHCIIpan 3.2^14^, an in silico HLA-peptide binding affinity calculation tool.

Since DQA1*05:03 is reportedly a disease-sensitive allele in Japanese patients, the *DQA1* and *DQB1* alleles analyzed were limited to those known to be present in the Japanese population based on the HLA Laboratory website data (https://hla.or.jp/med/frequency_search/en/haplo/). In addition, it is generally reported that DQB1*05 and DQB1*06 alleles form pairs only with the DQA1*01 alleles, and vice versa, so they were excluded from the analysis (Table 2). It should be noted that the output of this tool is not affected by the 2nd field (formerly 4-digit) of the *DQA1* allele, so we excluded its description. The immunodominant AQP4 peptides (7 peptides) and the non-immunodominant AQP4 peptides (24 peptides) are shown in Table 1.

**Table 2:** *DQA1* and *DQB1* alleles used for analysis with NetMHCIIpan 3.2.

DQA1 alleles	DQB1 alleles
*02:01	*02:01
*03:01	*02:02
*03:02	*03:01
*03:03	*03:02
*04:01	*03:03
*05:01	*04:01
*05:03	*04:02
*05:05	
*05:06	
*05:08	
*06:01	

The *HLA* alleles listed are those that have been confirmed in the Japanese population.

**Figure 3:**
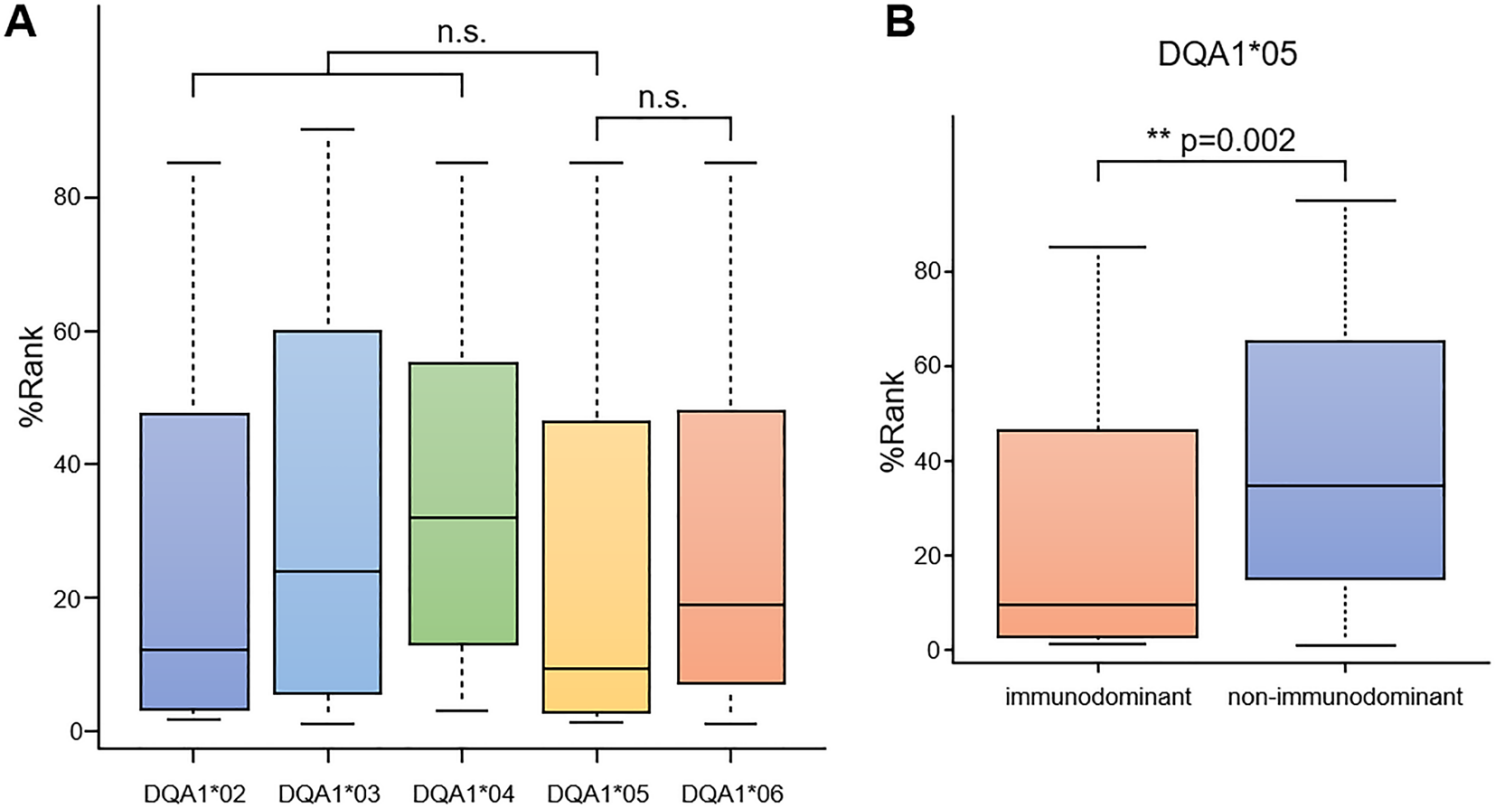
The binding affinity of aquaporin 4 (AQP4) peptides and HLA-DQ molecules (NetMHCIIpan 3.2). (**A**) Comparison of binding affinity among *HLA-DQA1* alleles binding to seven immunodominant AQP4 peptides. The y-axis values represent %Rank, i.e., the percentile of predicted binding affinity compared to a set of 200,000 random natural peptides that have the same amino acid length of the target peptides, thus lower %Rank showing higher binding affinity. Binding affinities were calculated for all combinations of the *DQA1* and *DQB1* alleles (Table 2) and the seven immunodominant AQP4 peptides (Table 1). (**B**) Binding affinity of immunodominant AQP4 peptides (7 peptides) and non-immunodominant AQP4 peptides (24 peptides) in the HLA-DQ molecule encoded by the DQA1*05 allele.

First, we calculated binding affinities for all combinations of the seven immunodominant AQP4 peptides (Table 1), the *HLA-DQA1* alleles and *HLA-DQB1* alleles (Table 2), and subsequently compared them among the *HLA-DQA1* alleles (Fig. 3A). In comparison with the other *HLA-DQA1* alleles, HLA-DQA1*05 showed no statistically significant difference. Next, focusing on HLA-DQA1*05, we calculated binding affinities for all the combinations of AQP4 peptide (Table 1) and *HLA-DQB1* alleles (Table 2), and then compared the calculated results between two groups of immunodominant AQP4 peptides and non-immunodominant AQP4 peptides (Fig. 3B). We found that HLA-DQA1*05 binds more potently to immunodominant AQP4 peptides than to non-immunodominant AQP4 peptides.

### The pMHC complex of DQA1*05:03 with immunodominant AQP4 peptides is energetically stable

**Figure 4:**
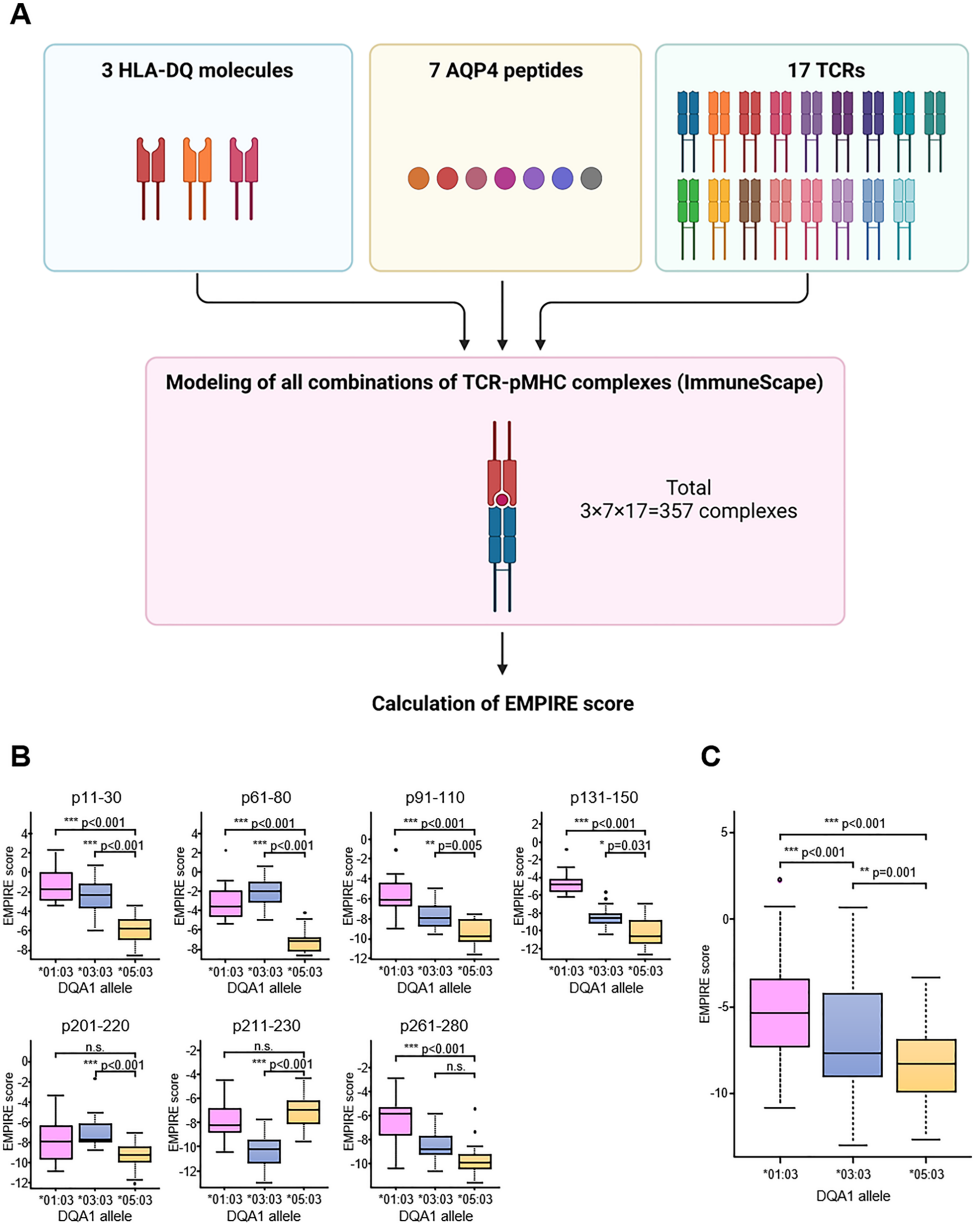
The energetic stability of pMHC complex. (**A**) Procedure for modeling and calculation of EMPIRE score using ImmuneScape. This schema was created with BioRender.com. (**B**, **C**) Comparison of energetic pMHC complex stability between three *HLA-DQA1* alleles binding. (**B**) EMPIRE score with each immunodominant AQP4 peptide. (**C**) Average EMPIRE score with all seven immunodominant AQP4 peptides.

Next, using ImmuneScape, a tool that is capable of modeling MHC, peptide, and T cell receptor (TCR) complexes^15^, we evaluated the energetic stability of the pMHC complex. All complexes were modeled using 17 distinct TCRs from the Protein Data Bank (PDB) (see the description in the Methods section). For all combinations of these *HLA-DQA1* alleles, AQP4 peptides, and TCRs, the energetic stability of each pMHC complex was evaluated by the EMPIRE (EMpirical Protein-InteRaction Energy) score function^16^ and compared among the *DQA1* alleles (Fig. 4A). Here, lower EMPIRE score represents higher energetic stabilization of pMHC.

**Figure 5:**
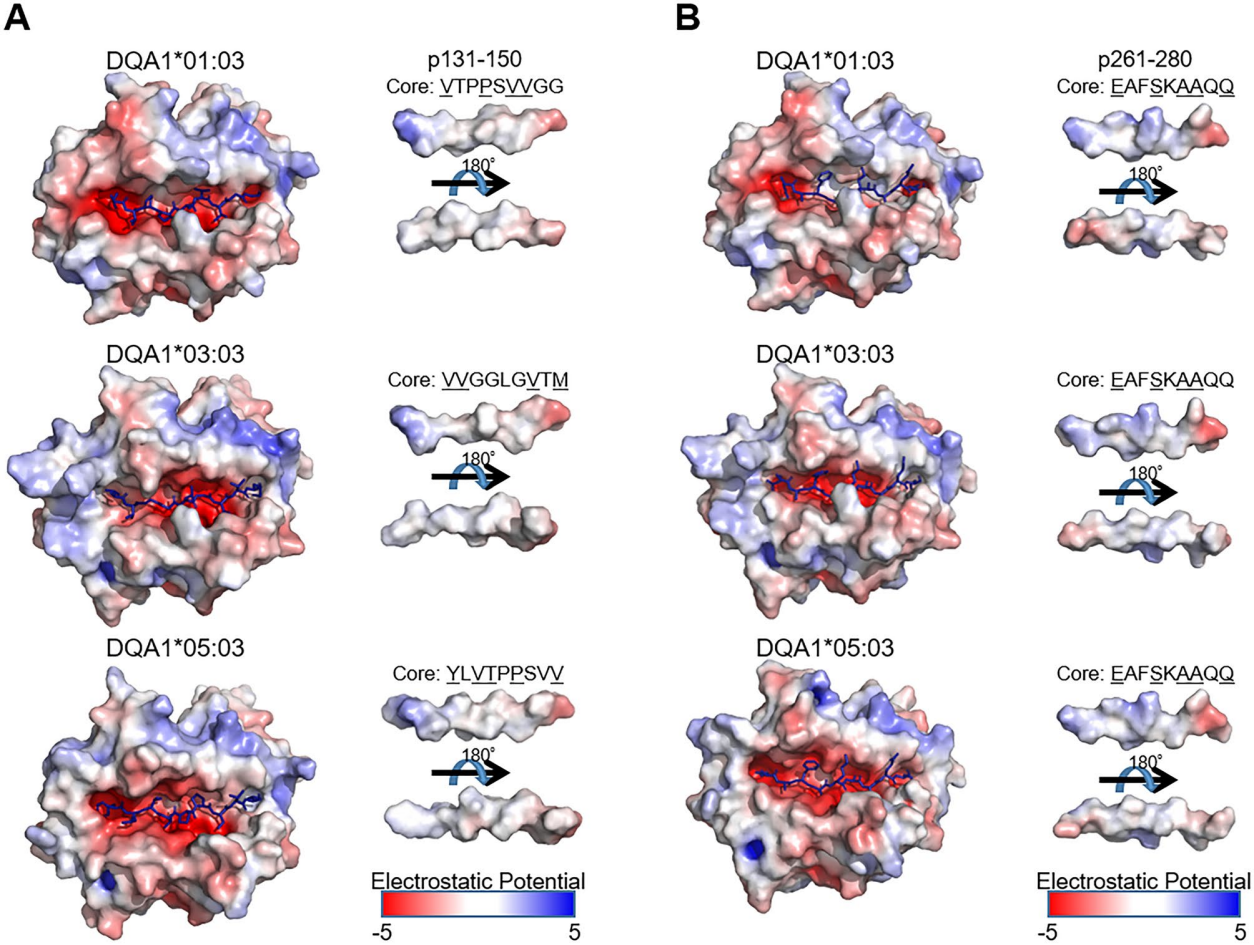
The modeling of pMHC complex. (**A**, **B**) The modeling of pMHC complex in three *DQA1* alleles binding to two AQP4 peptides; p131–150 (**A**) and p261–280 (**B**).

In 4 of the 7 immunodominant AQP4 peptides (p11–30, p91–110, p131–150 and p261–280), the energetic stabilization of pMHC was significantly higher in the order of HLA-DQA1*05: 03, DQA1*03:03 and DQA1*01:03 (Fig. 4B). In addition, when all seven AQP4 peptides were analyzed together, a significant difference in the degree of energetic stabilization was observed in the same order (Fig. 4C). Moreover, 3D structure modeling was performed using ImmuneScape on both p131–150 and p261–280 (Fig. 5A, B). In both models, the volume of the peptide cavity was smaller in HLA-DQA1*01:03 and DQA1*03:03 than in HLA-DQA1*05:03. It is well-established that conformational entropy contributes significantly to peptide-protein binding affinity^17,18^. Thus, we speculated that the larger binding cavity provided greater conformational entropy to the bound peptides, contributing to the lower EMPIRE energies. In the case of p131–150, the core peptide differed for each *HLA-DQA1* allele. Both HLA-DQA1*03:03 and DQA1*05:03 could bury a hydrophobic side-chain (methionine and valine, respectively) on the C-terminus, but only HLA-DQA1*05:03 could bury a large aromatic side-chain (tyrosine). This probably explains the better binding energy. In the case of p261–280, all three alleles were predicted to bind the same core peptide, and this peptide was predicted to bind with a similar conformation. However, the cavity of HLA-DQA1*05:03 was larger, thus allowing the anchor side-chains to assume different orientations for the N- and C-terminal residues.

In summary, our findings revealed that HLA-DQA1*05:03 is energetically more stable than other *HLA-DQA1* alleles when bound to the immunodominant AQP4 peptide, and according to the 3D structure models, it was further rationalized by the large volume of the peptide cavity of DQA1*05:03, possibly contributing favorably to the binding affinity of the pMHC complex.

## Discussion

Although several reports have been made on the disease-susceptible *HLA* alleles of NMOSD in Japanese, Southern Han Chinese, Asian, Latin American and European populations^19–25^, there was no common disease-susceptibility allele regardless of race. Meanwhile, a large-scale genome-wide association study was carried out on NMOSD patients of European ancestry in 2018, and the findings revealed that one of the single nucleotide polymorphisms that has the strongest association with NMOSD susceptibility was located in the *HLA-DQA1* gene, with DQA1*05:01 reported as one of the highest risk alleles^3^. Moreover, in 2019, Ogawa et al. performed next-generation sequencing on Japanese NMOSD patients and identified HLA-DQA1*05:03 as the most susceptible allele^4^. In Japan, unlike in Europe, the frequency of HLA-DQA1*05:03 in the population is much higher than that of HLA-DQA1*05:01, so the subtle differences in the findings between these two major genetic surveys may possibly have been due to genetic differences. In fact, the amino acid sequences of HLA-DQA1*05:03 and DQA1*05:01 differ by only one amino acid in the α2 domain (i.e., the amino acid sequences of the peptide binding clefts are identical), so it was considered that both of these two alleles might affect the pathophysiology of NMOSD via a similar mechanism.

It should be noted that there are many diseases, including autoimmune diseases, for which specific *HLA* alleles and haplotypes are reportedly strongly associated with the disease susceptibility or protection. However, few reports have focused on clarifying the specific pathogenic mechanism by which the disease-sensitive allele (or protective allele) promotes the onset of the disease. Moreover, only a few papers have focused on the effect of HLA cell surface expression. Pisapia et al. reported that the DQα1*05 chain and DQ1β*02 chain, which are known as risk alleles for celiac disease and type 1 diabetes, have significantly higher cell surface expression levels in B lymphoblastoid cell lines than non-risk alleles DQα chain and DQβ chain^26^.

It has been reported that the cell surface expression level of HLA molecules changes dramatically depending on the haplotype^27,28^. Miyadera et al. comprehensively measured the cell surface expression level of HLA-DQ molecules, and reported that it differs greatly depending on the combination of α and β chain that make up the HLA-DQ molecule^27^. Although HLA-DQA1*05:03 was not addressed in that study, the hierarchy of cell surface expression observed with other *HLA-DQA1* alleles was consistent with our findings. In addition, by identifying the responsible residues that regulate the cell surface expression through mutagenesis studies and association analysis, they demonstrated that the hierarchies are mainly formed by polymorphic sites that directly affect the interactions between the subunits or domains constituting the HLA molecules, or between HLA molecules and binding peptides. Furthermore, in a study by Kaur et al., the authors concluded that the high thermal stability of pMHC encoded by HLA-C*05 compared to HLA-C*07 resulting from variations in amino acid residues constituting the peptide binding groove of HLA-C molecules can explain the high cell surface expression of pMHC encoded by HLA-C*05^29^. What these two studies have in common is that the difference in cell surface expression of HLA molecules between alleles is independent of the mRNA level and intracellular total protein level of HLA molecules in HLA-expressing cells. This is consistent with the results of the transfection experiment we obtained in this study using the pcDNA3.1 (+) IRES GFP plasmid vector. Our findings revealed that the cell surface expression of the HLA-DQ molecule encoded by DQA1*05:03 was remarkably high, and that its molecular mechanism was due to the amino acid sequence of the HLA-DQ molecule itself.

After quantifying the cell surface expression level of HLA-DQ molecules in vitro, we examined the binding affinity of HLA-DQ and AQP4 peptides and the energetic stability of the complex in silico. Our findings indicated that the binding affinity between the HLA-DQ molecule encoded by DQA1*05:03 and the immunodominant AQP4 peptide was relatively high, and that the energetic stability of the complex was also higher than that of other *HLA-DQA1* alleles. In fact, the HLA-DR molecule encoded by the systemic sclerosis with anti-Topoisomerase 1 antibody (ATASSc)-associated alleles (HLA-DRB1*08:02, HLA-DRB1*11:01 and HLA-DRB1*11:04) or suspected allele (HLA-DRB5*01:02) is reported to have a high binding affinity for the immunodominant peptide of topoisomerase 1 self-protein (residues 349–368)^30^. Furthermore, it has been shown that high stability of the peptide-MHC class II complex broadens the clonotypic repertoire of antigen-specific CD4+ T cells, while low stability skews it into a restricted repertoire with high affinity TCRs for pMHC^31^. Based on these facts, the high binding affinity between the HLA-DQ molecule encoded by DQA1*05:03 and immunodominant AQP4 peptides and the stability of the complex may characterize the clonotypic repertoire of CD4+ T cells and induce the production of anti-AQP4 antibodies, thus resulting in the development of NMOSD.

There are certain limitations that should be taken into consideration in future studies. First, the cell surface expression level of HLA molecules was measured by a transient transfection assay on HEK293 cells, which may not reflect the expression profile of HLA-DQ molecules in APCs of actual NMOSD patients. Moreover, only HLA-DQB1*03:01 was paired with *DQA1* alleles, so there is no certainty that the results of this experiment can be reproduced with other *DQB1* alleles. In addition, we did not evaluate the effects of other alleles that are in a linkage disequilibrium with HLA-DQA1*05:03. Finally, the sensitivity and specificity of the structural models obtained in silico needs to be verified by further in vitro studies.

In conclusion, the findings in this study indicated the pathogenic roles of disease-susceptible *HLA* alleles in the development of NMOSD, an autoimmune disease characterized by the production of anti-AQP4 autoantibodies. Thus, we propose that the cell surface expression level of the HLA molecule, the binding affinity with the immunodominant AQP4 peptide, and the stability of the complex may play important roles in the pathophysiology of NMOSD.

## Methods

### Plasmids

All *HLA* expression vectors were generated at the Research Institute for Microbial Diseases, Osaka University, Suita, Japan. Complementary DNAs (cDNAs) prepared from pooled human peripheral blood mononuclear cells (3H Biomedical AB, Uppsala, Sweden) were cloned into the pME18S vectors. cDNA sequences for HLA class II were based on information contained in the Immunogenetics/HLA Database (http://www.ebi.ac.uk/imgt/hla/index.html).

### Construction of co-expression vectors of GFP and *HLA-DQA1* allele

To assess the cell surface expression of HLA-DQ molecules on HEK293 cells, we incorporated the HLA-encoding fragment of each *HLA-DQA1* expression vector (DQA1*01:03, DQA1*03:03 and DQA1*05:03) into the pcDNA3.1(+)IRES GFP, which was a gift from Kathleen L. Collins (Addgene plasmid # 51406; http://n2t.net/addgene:51406; RRID:Addgene_51406)^32^. Briefly, the fragment encoding the HLA-DQ α chain of each *HLA-DQA1* expression vector was amplified by polymerase chain reaction (PCR) using KOD FX (Toyobo Co., Ltd., Osaka, Japan). Nhe1 cleavage sequence was added to the 5'side of the forward primer and the Not1 cleavage sequence to the 5′ side of the reverse primer at the beginning of the start codon and stop codon of the sequence encoding the α chain, respectively. The PCR product was electrophoresed on an agarose gel, and the DNA fragment in the gel was extracted by use of the illustra™ GFX™ PCR DNA and Gel Band Purification Kit (Cytiva, Tokyo, Japan). It was ligated to pCR™-Blunt vector (Invitrogen™, Carlsbad, CA, USA) using DNA Ligation Kit Version 2.1 (Takara Bio, Inc., Shiga, Japan) according to the manufacturer's instructions. By ligating the cleaved fragment encoding the α chain obtained by cutting the synthesized pCR™-Blunt vector with Nhe1 and Not1 to the MCS of the pcDNA3.1 (+) IRES GFP plasmid vector, the GFP expression vectors that co-express the α chain of the HLA-DQ molecule and GFP were synthesized.

### Transient transfection of HLA plasmids in HEK293 cells

HEK293 cells were grown and maintained in 96-well culture plates in Dulbecco’s Modified Eagle’s Medium supplemented with 10% fetal bovine serum and 1% penicillin–streptomycin in a tissue culture incubator at 37 °C with 5% CO_2_. After reaching approximately 70% confluence, the cells were transfected with both the *HLA-DQA1* and the *HLA-DQB1* expression vectors using FuGENE^®^ 6 Transfection Reagent (Promega Corporation, Madison, WI, USA). At 48-h after transfection, they were used for flow cytometry analysis to evaluate the cell surface expression levels.

### Flow cytometry and antibodies

HLA-DQ cell surface expression levels and GFP were measured in transfected HEK293 cells using PE anti-human HLA-DQ Antibody (BioLegend^®^, San Diego, CA, USA) and HLA-DR/DP/DQ Monoclonal Antibody (WR18), PE (Invitrogen™) by FACSCanto™ II (BD Bioscience, San Jose, CA). All assays were performed three or more times independently for each *HLA-DQA1* and *DQB1* allele pair. These data were analyzed using the FlowJo software (BD Bioscience).

### Stimulating AQP4 peptides

We selected the following five immuodominant AQP4 peptides: AQP4 p61–80, p91–110, 131–150, 201–220 and 211–230. These peptides were commercially synthesized by GenScript^®^ Japan, Inc. (Tokyo, Japan) with purity > 95%. Sequences of these peptides were based on UniProt database (AQP4_HUMAN P55087-1). Each of these synthesized peptides was added to the culture medium of HLA expressing HEK293 cells at a concentration of 10 μg ml^−1^, and the cell surface expression level of HLA was evaluated by flow cytometry after culturing for 8 h.

### Construction of modified *HLA-DQA1* expression vectors with swapped sequences

Each *HLA-DQA1* expression vector (DQA1*01:03, DQA1*03:03 and DQA1*05:03) was cut into two fragments, one of which encoded the α1 domain and the other the α2, TM and CY domain. They were electrophoresed on an agarose gel and their DNA fragments were extracted with the illustra™ GFX™ PCR DNA and Gel Band Purification Kit. In accordance with the manufacturer's instructions, the fragment encoding the α1 domain and the fragment encoding the α2, TM and CY domains were exchanged and ligated respectively using the DNA Ligation Kit Version 2.1.

### Construction of mutant HLA-DQA1*05:03 expression vectors

We constructed four mutant *HLA-DQA1* expression vectors, each with one amino acid substitution in the α1 domain sequence from HLA-DQA1*05:03 to HLA-DQA1*01:03; V50E, Q53K, F54_R55insG and L75M. These mutant vectors were synthesized using QuikChange Lightning Site-Directed Mutagenesis Kit (Agilent Technologies, Santa Clara, CA, USA) according to the manufacturer's instructions.

### Measurement of binding affinity of AQP4 peptides and HLA-DQ molecules using NetMHCIIpan 3.2

Measurement of binding affinity of AQP4 peptides and HLA-DQ molecules was performed with the artificial neural network–based alignment method NetMHCIIpan 3.2 (http://www.cbs.dtu.dk/services/NetMHCIIpan-3.2/), as described previously. This system was generated by using an extended data set of quantitative MHC-peptide binding affinity data obtained from the Immune Epitope Database^33^ covering HLA-DR, HLA-DQ, HLA-DP and H-2 mouse molecules. Trained with these data, this system can predict binding affinity of any peptide for any MHC class II haplotype in silico. The output formats are IC50 and %Rank (percentile rank), and we adopted the latter in this study so that the values could be compared among different *HLA* haplotypes, as IC50 is defined as 50% binding inhibition concentration for standard peptide and the standard peptide varies depending on the *HLA* haplotype.

### Energetic stability analysis and 3D models of pMHC complex

The pMHC complexes were modeled for each combination of 7 immunodominant AQP4 peptides (p11–30, p61–80, p91–110, p131–150, p201–220, p211–230 and p261–280), 3 *HLA-DQA1* alleles (DQA1*01:03, DQA1*03:03 and DQA1*05:03), and 1 *DQB allele* (DQB1*03:01) using a customized version of the ImmuneScape^15^ workflow. ImmuneScape requires not only MHC and epitope, but also TCR sequences; the program then returns a 3D model of the TCR-peptide-MHC complex. In order to ensure that the results did not depend on the chosen TCR, all complexes were modeled with 17 different TCRs. These TCRs were based on ImmuneScape's structure template library, which is a subset of experimentally verified structures from the PDB. The TCR sequence pairs (combined alpha and beta chains) were clustered using cd-hit with a similarity cutoff of 0.8 to remove redundancy. The resulting 17 cluster representatives were used for structural modeling, with the obtained EMPIRE energy results averaged across all 17 TCRs. ImmuneScape extracts 9-residue epitope cores from the full-length input peptides using NetMHCIIpan^14^ predictions. For each TCR-epitope-MHC model, the binding energies (MHC alpha and beta vs epitope) were evaluated using EMPIRE^16^, which computes a statistics-based protein–protein binding energy.

### Statistical analysis

All statistical analyses were performed by the Kruskal–Wallis test followed by Steel–Dwass multiple comparisons using EZR statistical software (Saitama Medical Center, Jichi Medical University, Saitama, Japan), which is a graphical user interface for R commander (The R Foundation for Statistical Computing, Vienna, Austria) designed to add statistical functions frequently used in biostatistics^34^. Data given in a box plot show the median (horizontal line within the box), the 25th and 75th percentile (boundary of the box), and the 10th and 90th percentile (whiskers above and below the box). Data points not included in this range are indicated by dots as outliers.

## Supplementary Information

Supplementary Figure S1.

## References

[1] Jacob A (2013). Current concept of neuromyelitis optica (NMO) and NMO spectrum disorders. J. Neurol. Neurosurg. Psychiatry.

[2] Lennon PVA (2004). A serum autoantibody marker of neuromyelitis optica: Distinction from multiple sclerosis. Lancet.

[3] Estrada K (2018). A whole-genome sequence study identifies genetic risk factors for neuromyelitis optica. Nat. Commun..

[4] Ogawa K (2019). Next-generation sequencing identifies contribution of both class i and II HLA genes on susceptibility of multiple sclerosis in Japanese. J. Neuroinflammation.

[5] Varrin-Doyer M (2012). Aquaporin 4-specific T cells in neuromyelitis optica exhibit a Th17 bias and recognize Clostridium ABC transporter. Ann. Neurol..

[6] Snapper CM, Paul WE (1987). Interferon-γ and B cell stimulatory factor-1 reciprocally regulate Ig isotype production. Science.

[7] Toellner KM (1998). T helper 1 (Th1) and Th2 characteristics start to develop during T cell priming and are associated with an immediate ability to induce immunoglobulin class switching. J. Exp. Med..

[8] Sagan SA (2016). Tolerance checkpoint bypass permits emergence of pathogenic T cells to neuromyelitis optica autoantigen aquaporin-4. Proc. Natl. Acad. Sci. USA.

[9] Matsuya N (2011). Increased T-cell immunity against aquaporin-4 and proteolipid protein in neuromyelitis optica. Int. Immunol..

[10] Vaknin-Dembinsky A (2016). T-cell responses to distinct AQP4 peptides in patients with neuromyelitis optica (NMO). Mult. Scler. Relat. Disord..

[11] Hofer LS (2020). Comparative analysis of T-cell responses to aquaporin-4 and myelin oligodendrocyte glycoprotein in inflammatory demyelinating central nervous system diseases. Front. Immunol..

[12] Watanabe N (2017). A cell-based high-throughput screening assay system for inhibitor compounds of antigen presentation by HLA class II molecule. Sci. Rep..

[13] Höpner S (2006). Small organic compounds enhance antigen loading of class II major histocompatibility complex proteins by targeting the polymorphic P1 pocket. J. Biol. Chem..

[14] Jensen KK (2018). Improved methods for predicting peptide binding affinity to MHC class II molecules. Immunology.

[15] Li S (2019). Structural modeling of lymphocyte receptors and their antigens. Methods Mol. Biol..

[16] Liang S, Liu S, Zhang C, Zhou Y (2007). A simple reference state makes a significant improvement in near-native selections from structurally refined docking decoys. Proteins.

[17] Forman-Kay JD (1999). The ‘dynamics’ in the thermodynamics of binding. Nat. Struct. Biol..

[18] Killian BJ (2009). Configurational entropy in protein-peptide binding: Computational study of Tsg101 ubiquitin E2 variant domain with an HIV-derived PTAP nonapeptide. J. Mol. Biol..

[19] Guimarães Brum D (2010). HLA-DRB association in neuromyelitis optica is different from that observed in multiple sclerosis. Mult. Scler..

[20] Wang H (2011). HLA-DPB1*0501 is associated with susceptibility to anti-aquaporin-4 antibodies positive neuromyelitis optica in Southern Han Chinese. J. Neuroimmunol..

[21] Zéphir H (2009). Is neuromyelitis optica associated with human leukocyte antigen?. Mult. Scler..

[22] Alvarenga MP (2021). Neuromyelitis optica is an HLA associated disease different from Multiple Sclerosis: a systematic review with meta-analysis. Sci. Rep..

[23] Matsushita T (2009). Association of the HLA-DPB1*0501 allele with anti-aquaporin-4 antibody positivity in Japanese patients with idiopathic central nervous system demyelinating disorders. Tissue Antigens.

[24] Yoshimura S (2013). Distinct genetic and infectious profiles in Japanese neuromyelitis optica patients according to anti-aquaporin 4 antibody status. J. Neurol. Neurosurg. Psychiatry.

[25] Matsushita T (2020). Genetic factors for susceptibility to and manifestations of neuromyelitis optica. Ann. Clin. Transl. Neurol..

[26] Farina (2019). HLA-DQA1 and HLA-DQB1 alleles, conferring susceptibility to celiac disease and type 1 diabetes, are more expressed than non-predisposing alleles and are coordinately regulated. Cells.

[27] Miyadera H, Ohashi J, Lernmark Å, Kitamura T, Tokunaga K (2015). Cell-surface MHC density profiling reveals instability of autoimmunity-associated HLA. J. Clin. Invest..

[28] Tregaskes CA (2016). Surface expression, peptide repertoire, and thermostability of chicken class I molecules correlate with peptide transporter specificity. Proc. Natl. Acad. Sci. USA.

[29] Kaur G (2017). Structural and regulatory diversity shape HLA-C protein expression levels. Nat. Commun..

[30] Kongkaew S (2019). Interactions of HLA-DR and topoisomerase I epitope modulated genetic risk for systemic sclerosis. Sci. Rep..

[31] Baumgartner CK, Ferrante A, Nagaoka M, Gorski J, Malherbe LP (2010). Peptide-MHC Class II complex stability governs CD4 T cell clonal selection. J. Immunol..

[32] Schaefer MR (2008). A novel trafficking signal within the HLA-C cytoplasmic tail allows regulated expression upon differentiation of macrophages. J. Immunol..

[33] Vita R (2019). The immune epitope database (IEDB): 2018 update. Nucleic Acids Res..

[34] Kanda Y (2013). Investigation of the freely available easy-to-use software ‘EZR’ for medical statistics. Bone Marrow Transplant..

