# Supplementary material for: High cell surface expression and peptide binding affinity of HLA-DQA1*05:03, a susceptible allele of neuromyelitis optica spectrum disorders (NMOSD)
Source: Sci Rep. 2022 Jan 7;12:106. doi: 10.1038/s41598-021-04074-1 (PMC8742014; doi:10.1038/s41598-021-04074-1)
Supplement: Supplementary file 1 — Supplementary Figure S1. [file 41598_2021_4074_MOESM1_ESM.pdf]

Supplemental Information (Supplemental Figure)

Title

High cell surface expression and peptide binding affinity of *HLA-DQA1*05:03*, a susceptible allele of neuromyelitis optica spectrum disorders (NMOSD)

Authors

Shohei Beppu[1], Makoto Kinoshita[1*], Jan Wilamowski[2], Tadahiro Suenaga[3], Yoshiaki Yasumizu[1,4,5], Kotaro Ogawa[1], Teruyuki Ishikura[1], Satoru Tada[1], Toru Koda[1], Hisashi Murata[1], Naoyuki Shiraishi[1], Yasuko Sugiyama[1], Keigo Kihara[1], Tomoyuki Sugimoto[6], Hisashi Arase[7,8], Daron M. Standley[2,9], Tatsusada Okuno[1*] & Hideki Mochizuki[1,5]

Affiliations

[1]Department of Neurology, Graduate School of Medicine, Osaka University, 2-2 Yamadaoka, Suita, Osaka 565-0871, Japan. [2]Department of Genome Informatics, Genome Information Research Center, Research Institute for Microbial Diseases, Osaka University, 3-1 Yamadaoka, Suita, Osaka 565-0871, Japan. [3]Department of Microbiology, Fukushima Medical University, 1 Hikariga-oka, Fukushima, Fukushima 960-1295, Japan. [4]Department of Experimental Immunology, WPI Immunology Frontier Research Center, Osaka University, 3-1 Yamadaoka, Suita, Osaka 565-0871, Japan. [5]Integrated Frontier Research for Medical Science Division, Institute for Open and Transdisciplinary Research Initiatives (OTRI), Osaka University, 2-2 Yamadaoka, Suita, Osaka 565-0871, Japan. [6]Graduate School of Data Science, Shiga University, 1-1-1 Banba, Hikone, Shiga 522-8522, Japan. [7]Department of Immunochemistry, Research Institute for Microbial Diseases, Osaka University, 3-1 Yamadaoka, Suita, Osaka 565-0871, Japan. [8]Laboratory of Immunochemistry, WPI Immunology Frontier Research Center, Osaka University, 3-1 Yamadaoka, Suita, Osaka 565-0871, Japan. [9]Systems Immunology Laboratory, WPI Immunology Frontier Research Center, Osaka University, 3-1 Yamadaoka, Suita, Osaka 565-0871, Japan.

[*] Corresponding authors: Makoto Kinoshita and Tatsusada Okuno, Department of Neurology, Osaka University Graduate School of Medicine, 2-2 Yamadaoka, Suita, Osaka 565-0871, Japan. Tel.: +81-6-6879-3571, Fax.: +81-6-6879-3579

E-mail: mkinoshita@neurol.med.osaka-u.ac.jp (M.K.), okuno@neurol.med.osaka-u.ac.jp (T.O.)

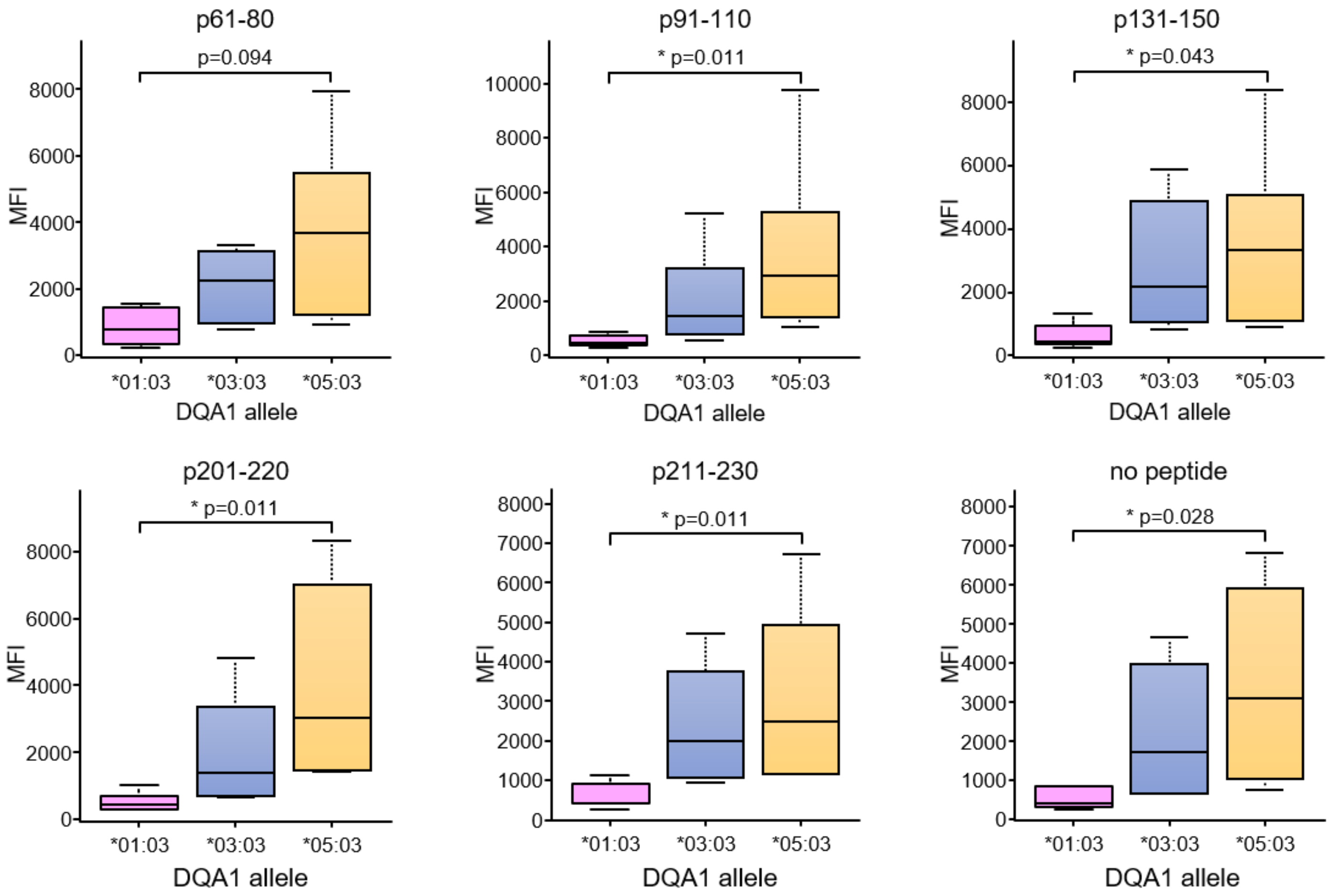

Supplemental Figure 1. Expression of HLA-DQ molecules co-cultured with AQP4 peptides. The expression levels of HLA-DQ molecules on the surface of transfected HEK cells when co-cultured with five immunodominant AQP4 peptides or no peptide, respectively.
